# The metabolic profile of Asinara (albino) and Sardo donkeys (pigmented) (*Equus asinus* L., 1758) points to unequivocal breed assignment of individuals

**DOI:** 10.7717/peerj.9297

**Published:** 2020-07-08

**Authors:** Maria Grazia Cappai, Corrado Dimauro, Giovanni Paolo Biggio, Raffaele Cherchi, Francesca Accioni, Flavia Pudda, Gianpiero Boatto, Walter Pinna

**Affiliations:** 1 Research Unit for Animal Nutrition, Department of Veterinary Medicine, University of Sassari, Sassari, Italy, Italy; 2 Department of Agriculture, University of Sassari, Sassari, Italy; 3 Department of Research and Development of Equine Production, AGRIS of the Autonomous Region of Sardinia, Cagliari, Italy; 4 Department of Chemistry and Pharmacy, University of Sassari, Sassari, Italy; 5 FORESTAS, Centro di recupero della Fauna Selvatica di Bonassai, Bonassai Olmedo, Italy

**Keywords:** Albinism, Mediterranean maquis, Sun radiation, Vitamin E

## Abstract

This study pointed to explore if variations in circulating levels of metabolites in the blood stream of no. 25 feral donkeys occur in view of the different coat color between specimens of Asinara (albino, no. 8) vs. Sardo (dun-grey, no. 17) breed. All individuals involved in this investigation are living in the nature, at Mediterranean latitudes and roam in the same areas all over the National Park of Capo Caccia, where they feed on spontaneous vegetation sources. The study was conducted during the positive photoperiod of the boreal hemisphere (peak in the month of June, 2019) to maximize the effect of exposure to the natural sun radiation and thus elicit the coping ability of albino (Asinara) in comparison with pigmented donkeys (Sardo). The biochemical profile of all donkeys was used in a Discriminant Analysis (DA) to explore if circulating levels of metabolites could point to metabolic markers for breed assignment of individuals following a canonical discriminant analysis (CANDISC). The biochemical investigation included also the determination of the circulating Vitamin E (alpha tocopherol, α-TOH), as an essential biologically active compound involved in antioxidant mechanisms, and its respective status (circulating α-TOH to total triglycerides and total cholesterol ratio). In the CANDISC, the distance between the two breeds was not significant. However, it pointed to different metabolites (UREA, total protein, total triglycerides, Zn) capable of describing biochemical patterns on each respective breed (Asinara vs. Sardo). The multivariate analysis DA carried out using 22 metabolites correctly assigned individuals to the two breeds in the 100% of cases. In view of such metabolic background, circulating α-TOH found in the bloodstream of Asinara vs. Sardo donkeys under free grazing conditions turned out to reach similar values (2.114 vs. 1.872 µg/ml, respectively, *p* = 0.676). It is worth noting that significant differences were observed as to circulating lactate dehydrogenase (LDH, *p* = 0.022) levels, in association with increased creatine phosphokinase (CPK, *p* = 0.076), both above the upper limit of the physiological range reported in other donkey breeds, and found in the totality of Asinara (albino) donkeys solely, still apparently clinically healthy.

## Introduction

The biochemical profile of feral animals represents a useful tool to understand how the organism responds to a series of external and internal stimuli. In particular, when animals live in the wild, blood biochemical analysis can allow to study the balance between all those factors for the maintenance of homeostasis or to reveal potential perturbations, if any. Moreover, particular metabolic conditions can provide information on the capability and extents of adaptation and coping with the environment. The Asinara donkey is a charismatic animal, originating and living on Asinara Island, in the Italian Autonomous Region of Sardinia, in the Mediterranean Sea (N 41°4′0.012″, E 8°16′0.012″, 51.9 km^2^). All specimens of this feral breed display to possess a peculiar phenotype, associated with a form of oculo-cutaneous albinism of type 1 (OCA1) (Utzeri et al., 2015; Cappai et al., 2015, 2017). Albinism is a condition characterized by the partial or complete lack of pigment in teguments, due to a genetically encoded biochemical impair of the melanogenic process (Okulicz et al., 2003; Grønskov, Ek & Brondum-Nielsen, 2007; Prado-Martinez et al., 2013).

The production of melanin pigments (eumelanin and pheomelanin) is elicited by external stimuli and ruled by the hormonal control of the pituitary gland, as a consequence of genetically encoded biochemical patterns. This being said, the production melanin forms, the dilution and overlap in certain region of the body result in the phenotype of the coat color of mammals (Sponenberg & Bellone, 2017). Albino phenotype is a rare occurrence, nevertheless it is spread across the animal kingdom, from reptiles to amphibians, fish, birds and mammals (Witkop, 1971; Oetting, Brilliant & King, 1996; Potterf et al., 1998; Oetting, 2000). Commonly, albinism is narrowed to individual level, but, in the case of Asinara donkeys, such phenotype is spread to all individuals of the breed, as a worldwide unique acknowledged breed of feral donkeys, freely grazing in the National Park of Asinara Island. Groups of specimens are also distributed in other regional Parks and reserves and are property of the Autonomous Region of Sardinia Isle. Few Asinara donkeys are also kept in other national Parks, The Asinara donkeys seem to have to branched out of Sardo donkeys (Pinna et al., 1998; Cosseddu et al., 2001), for which a common ancestor was identified. In Asinara donkeys, the impair of the melanogenic process (inactive tyrosinase, Cappai et al., 2015) is due to a missense mutation (Utzeri et al., 2015) in the gene encoding for the enzyme tyrosinase involved in the melanogenic process, fixed throughout generations under geographic isolation. The lack of pigment in the coat, skin, iris and natural opens in albino feral animals at Mediterranean latitudes may represent a metabolic challenge, following the excess of exposure to sun radiation, particularly during the hot season when light hours exceed dark hours. In fact, the augmented need of protection of external tissue from UV damage could pose a challenge to endogenous antioxidant systems, when natural pigments for coping with sun radiation are lacking.

Among diet derived antioxidants, fat soluble compounds of vitamin E group are abundant in vegetation. Browsing and grazing animals can intake large amounts of Vitamin E from fresh vegetal sources. Vitamin E accounts a group of fat soluble compounds involved in several biological processes in the animal body, important for health maintenance (Finno & Valberg, 2012). Alfa-tocopherol (α-TOH) represents one of the most biologically active forms of vitamin E group (DellaPenna & Pogson, 2006; Mustacich, Bruno & Traber, 2007). Isomers of vitamin E cannot be synthesized de novo in the animal body, thus circulating α-TOH determined in the bloodstream of animals derives from the diet. It was established that tocopherols are the most abundant isoforms of Vitamin E in leaves, whereas tocotrienols (among other isoforms of Vitamin E) are chiefly found in seeds (DellaPenna & Pogson, 2006). Though tocopherols can be found in plant seeds too, γ-tocopherol is abundantly synthesized, whereas α-TOH is produced only residually. Levels of circulating α-TOH in the bloodstream of free ranging animals may reflect leaf-based natural diets, especially for grazers, like equines. The content of natural α-TOH dramatically decreases in harvested feed, because fat soluble vitamins are in general intrinsically labile (light and heat/cold sensitive). In addition, vitamin E synthesis in plants broadly varies, according to plant species and season (higher during spring-summer than during fall-winter) (Sattler et al., 2003). As for the donkey, only a few contributions report detailed effects of the dietary regime on comparative biochemical profile and health conditions, with emphasis on specific nutrient deficiencies (Chiofalo et al., 2005; Cappai, Picciau & Pinna, 2013; Cappai et al., 2017; Girardi et al., 2013; Valle et al., 2017, 2018). In fact, very little is known about reference intervals of circulating parameters for this species (Zinkl et al., 1990; French & Patrick, 1995; Folch, Jordana & Cuenca, 1997; Mori et al., 2003; Caldin et al., 2005; Bana et al., 2011; Girardi et al., 2013; Laus et al., 2015; Burden et al., 2016; Da Silva et al., 2018; Santos et al., 2018; De Palo et al., 2018; Gloria et al., 2018; Barrio, Rickards & Thiemann, 2019). The interpretation of baseline levels of metabolites in free grazing donkeys through a comparative approach between albino and pigmented breeds is not reported in the present literature, to the best of our knowledge. It could be argued that albino Asinara donkeys may display peculiar metabolic profile, worth of being investigated.

The aims of the present study were to determine if differences may exist in values of circulating metabolites, which might be able to resume the breed specific metabolic fingerprint; to ascertain if the metabolic profile explored can be used to assign individuals to the two breeds (albino vs. pigmented coat); and, finally, to establish if the vitamin E status can be comparable between animals of the two breeds.

## Materials and Methods

The present investigation was approved by the Ethic Committee Organismo Preposto al Benessere e alla Sperimentazione Animale (OPBSA) of the University of Sassari, no. 101801.

**Figure 1:**
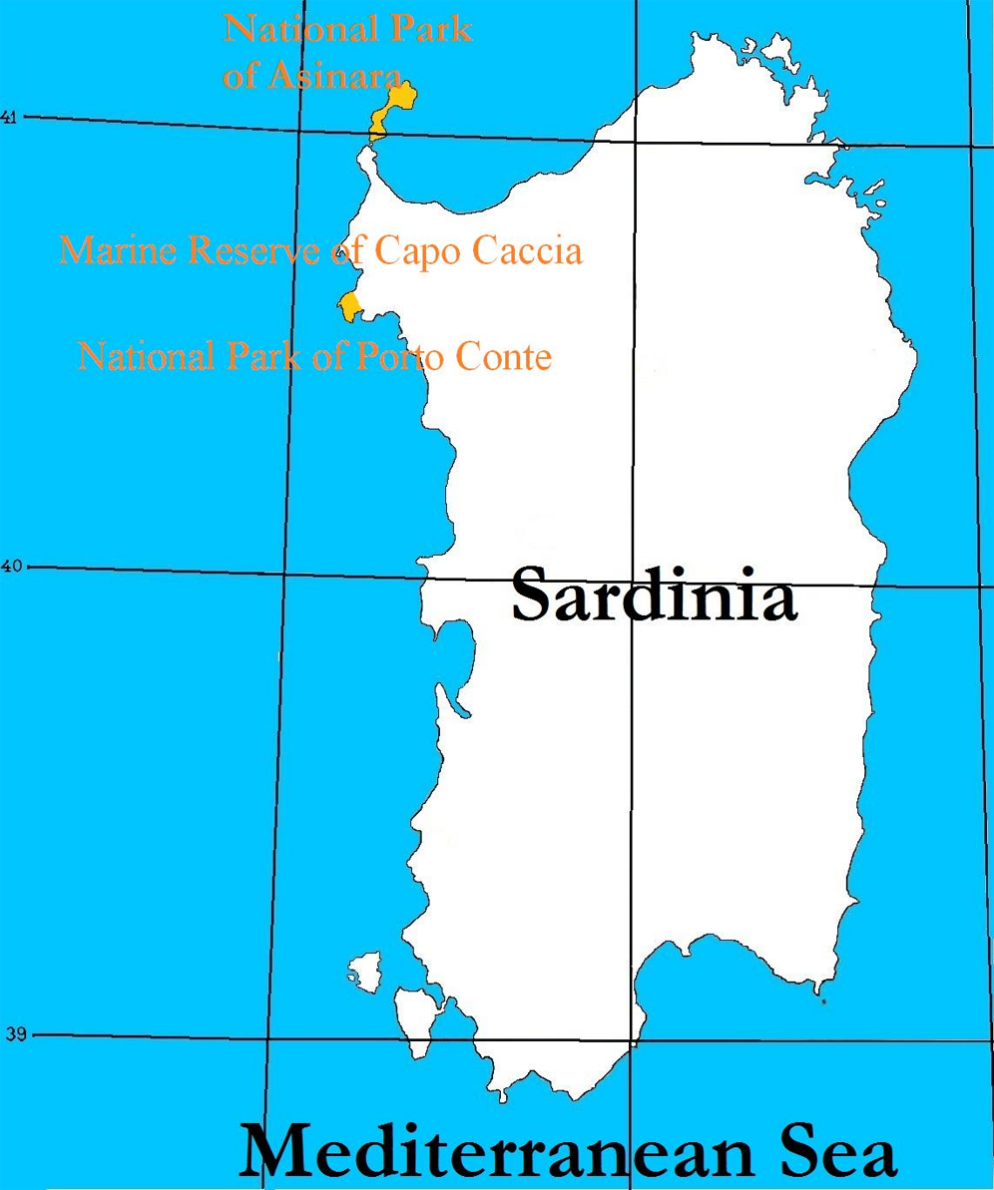
Map of Sardinia and latitudes of Asinara island where the National Park and Marine Reserve of Asinara is established, Capo Caccia and Porto Conte Parks (in yellow). The Asinara island and Capo Caccia promontory are colored in yellow.

**Figure 2:**
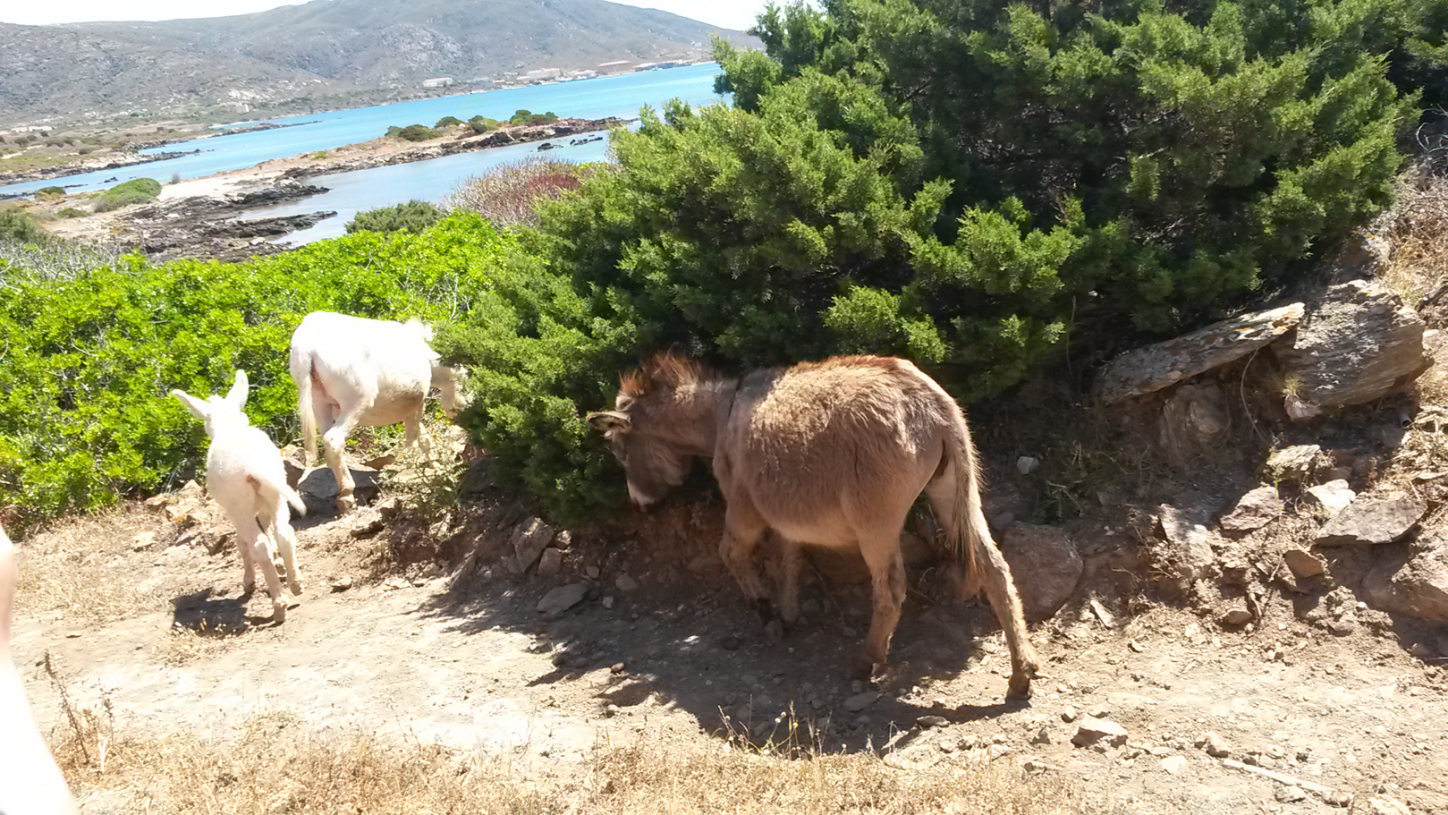
Asinara (jenny with foal) and Sardo (jenny) donkeys in the natural Mediterranean maquis of Asinara Park. Albino and pigmented donkeys roam in same areas in small groups and feed on spontaneous vegetation, represented by Mediterranean macchia and garrigue.

The study was carried out on animals of Asinara breed, derived by the Asinara Island in which they originate (N 41°4′0.012″, E 8°16′0.012″, 51.9 km^2^ in Sardinia, Italy), established as a National Park (Official Gazzette of the Italian Republic, 1997) and Marine Reserve (Official Gazzette of the Italian Republic, 2002) of the Autonomous Region of Sardinia (Italy), in the Mediterranean Sea (Fig. 1). The population of Asinara donkeys on the Asinara island is estimated to account 140 individuals. The National Registry of Local Minor Equine Breeds, Association of Italian Breeders (Associazione Italiana Allevatori, AIA) (2016) accounted 294 specimens, for which a critical conservation status is acknowledged. Feral Sardo breed donkeys (Fig. 2) live on the isle of Asinara and live in small groups (in total Sardo donkeys on the isle of Asinara are less than 250) with Asinara donkeys, as well as in other parks and reserves of the Autonomous Region of Sardinia, like the Capo Caccia Marine Reserve and Porto Conte Park, in the promontory of Capo Caccia area.

The investigation involved a total of 25 adult donkeys (age: between 5 and 6 years, established from records of the Park), of which no. 12 stallions and no. 13 jennies. Asinara (no. 8) and Sardo (no. 17) breed donkeys were enrolled to achieve similar sex ratio (1:1). All animals were electronically identified (EID, EU Regulation 2015/262) and recorded in the Official Register of the albino donkey of Asinara and Sardo breeds (Ministerial Decree 27/7/1990), respectively. The proportion of specimens from each breed was established to be representative of populations of Asinara and Sardo donkeys, namely hundreds and thousands under actual condition in Sardinia, respectively. The number of heads is checked and updated every year. Sanitary surveillance is also carried out by the Veterinary Services of the local District, for the control of equine infectious diseases. Wild and domestic equines undergo serological tests to monitor and control the presence of the Equine Infectious Anaemia (EIA, diagnosed by means of Coggins test, mandatory for horses, donkeys, mules and hinnies) and therefore they are captured for temporary captive period in facilities of parks and reserves of the Region, like in this case in Porto Conte Park. Asinara and Sardo donkeys are tame animals (Fig. 2) and are used to human contact as they represent the attraction of Parks. Approaching animals in the nature is not difficult. On blood sampling for serological analysis, one serum aliquot was used to determine the general biochemical profile of all donkeys enrolled. In the light of the focus of this investigation, circulating α-TOH was also determined on same samples, without requiring further manipulations of animals. Each donkey had free access to same natural areas. On blood sampling, donkeys underwent the nutritional assessment according to Cappai, Picciau & Pinna (2013).

### Sampling methods and analytical procedures

Blood sampling coincided with the peak of the positive photoperiod in the boreal hemisphere and blood samples of animals screened for serological purposes were collected in the month of June 2019 for the purposes of this investigation. Sampling was carried out over 5 days to allow the screening of all the groups, undergoing in field identification control and serological checking schedule to monitor and control the presence of EIA by the veterinary services. Whole blood was drawn by the puncture of the jugular vein into vacuum tubes (Vacutainer nine ml, Vacutainer Systems Europe; Becton Dickinson, Meylan Cedex, France) which were filled to reach proper volume, before storage in the upright position and cooling to 4 °C. For this purpose, groups of animals were gathered in a paddock by the personnel of the Park. Animals were induced to step into a corridor with the use of mobile fences, leading to a horse stock. All animals underwent a same protocol and were manipulated in respect of animal welfare, for the sole moment needed for blood sampling and EID code checking. All animals were immediately released, when all procedures were terminated.

Individual tubes were covered with tin-foils to protect blood from light and classified with individual labels. Tubes were held in the upright position through polystyrene cases and kept in a refrigerated bag during the transport of samples to the laboratory.

All laboratory procedures were started on whole blood within 6 h of collection, which is an established protocol internally developed on the basis of international reference, aiming to minimize statistical differences in hematological determination, over time. In field and laboratory protocols for collection, storage and analyses of blood were carried out keeping samples in the dark, to avoid photo-degradation of α-TOH. Individual sera were screened to explore organ function and assess overall metabolic conditions. Prior to chemical analyses, samples were centrifuged at 1,500 g at 4 °C degrees, for 10 minutes. Two aliquots (two ml) of each serum sample were stored in sterile vials and frozen at −20 °C until further analysis. All the samples were analyzed within one week of sampling moment, through an automatic light-protected biochemical analyzer (Mindray BS-200, Shenzhen, China). Concentration in blood serum of ubiquitous intermediate metabolites, enzymes, nutrients and macro-minerals (ALT, alanine transaminase; AST, aspartate aminotransferase; γ-GT, gamma-glutamyl transferase; CREA, creatinine; Urea; TBil, total bilirubine; TP, total protein; TC, total cholesterol; TG, total triglycerides; GLU, glucose; AMY, amylase; LIPA, Lipase; LDH, lactate dehydrogenase; CPK, creatine phosphokinase; ALP, alkaline phosphatase; Ca, calcium; P, phosphorus; Zn, Zinc; Cu, copper; Na, sodium; Cl, chloride; Mg, magnesium) was determined.

For the determination of α-TOH, high pressure liquid chromatography coupled with an ultraviolet detector (HPLC-UV) was carried out. All standards and solvents were purchased from Sigma–Aldrich (Milan, Italy). Stock solution (1 mg/ml) of α-TOH was prepared in chloroform/methanol (50/50). For the calibration curve, standard stock solutions were diluted with methanol and kept frozen at −20 °C, protected from light. Serum levels of α-TOH was measured at 280 nm. Chromatographic separation was carried out on a Waters Symmetry C18 column (4.6 × 150 mm, particle size 5 μm, Waters, Milford, MA, USA). The injection volume was 20 μl. The mobile phases used were acetonitrile/ methanol/ Milli-Q water (64.5/33/2.5) at 1 ml/min. Data were acquired and processed by Breeze Software (Waters, Milford, MA, USA). Samples were prepared as follows: 0.3 ml of serum was vortexed with 0.6 ml of acetonitrile and centrifuged at 3,500×*g* at 4 °C for 10 min. The supernatant was dried under a stream of nitrogen and the residue was reconstituted in 0.15 ml of mobile phase (Biesalski et al., 1986; Milne & Botnen, 1986; Ganière-Monteil et al., 1994; Levent et al., 2013; Gershkovich et al., 2014).

### Calculations and statistical methods

Circulating levels of metabolites and baseline concentrations of α-TOH in the blood serum of donkeys were analyzed and interpreted according to species-specific range values reported in the literature. Data were analyzed by a one way-ANOVA, for the comparison of averages determined in specimens of the two breeds (Asinara vs. Sardo). In addition, the values were analyzed through a multivariate statistical analysis by means of a discriminant analysis (DA) to see if it was possible to assign animals to the two breeds on the basis of individual metabolic profile. A canonical discriminant analysis (CANDISC) was then developed to detect which metabolites more act in separating the two breeds. All procedures were carried out through SAS 9.1 (Cary, Inc., Rural Hall, NC, USA). The statistic significance was set for *p*-value < 0.05.

## Results

All animals involved in the trial appeared healthy. No specific clinical signs could be pointed out in specimens of both breeds, except for skin redness involving ear tips, ocular contour (showing signs of epiphora) and backline in all Asinara donkeys, considered as common findings for the breed. On nutritional assessment of visually inspected tissues, adequate muscular condition and normal skeletal morphology were diagnosed in all animals. Adequate body condition (BCS, based on a 5-points scale, 1 = emaciation to 5 = obesity) was scored in both in Asinara and Sardo donkeys (3.25 ± 0.15 vs. 3.50 ± 0.10, respectively).

**Figure 3:**
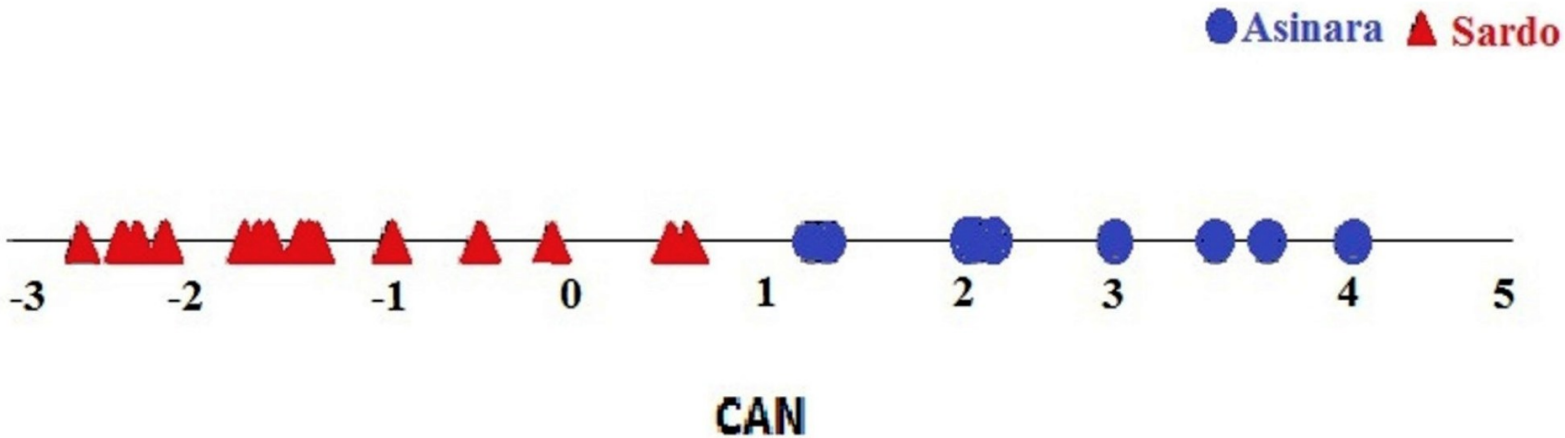
Plot of individual assignment to breeds at CANDISC. Blue symbols represent Asinara donkeys and red symbols represent Sardo donkeys. The sharp separation of blue and red symbols indicate the assignment of individuals to respective breeds according to coat colors, based on levels of circulating metabolites (22 explored). The assignment appears unequivocal due to no overlapping between blue and red symbols.

The totality of 23 serum samples collected from each donkey enrolled turned out to be diagnostic. Blood serum concentration of the 22 metabolites explored were determined on each individual sample. Biochemical profiles of specimens enrolled in this screening trial did not point to significant differences between breeds. Metabolites explored for organ function dropped within the physiological range reported for other donkey breeds (Laus et al., 2015; Burden et al., 2016), except for lactate dehydrogenase (LDH *p* = 0.022) and creatine phosphokinase (CPK *p* = 0.076), as reported in Table 1. The DA (the Box’s *M* test was not significant *p* > 0.10) pointed to a 100% successful assignment of individuals to the respective breed on the basis of circulating values of metabolites, like reported in the plot (Fig. 3). Blue symbols in the plot indicate the Asinara breed individuals, whilst red symbols identify Sardo breed donkeys. It can be seen that the distinction of individuals is sharp and no overlap can be pointed out. Despite the Hotelling’s test was non significant (Table 2), probably due to the lower number of samples from Asinara donkeys in comparison of Sardo ones, the assignment of donkeys appears unequivocal.

**Table 1:** Biochemical parameters of donkeys (Asinara *vs*. Sardo) in blood serum collected during positive photoperiod. Values are expressed as means and pooled standard deviation.

Breed	Asinara albino	Sardo pigmented	pooled-SD	*p*-Value
Coat Animals (no.)	8	17		
Metabolites				
GLU (mg/dl)	52.1	62.3	26.6	0.375
TG (mg/dl)	72.4	83.6	35.6	0.462
TP (g/l)	78.6	78.6	17.9	1.000
TC (mg/dl)	80.5	104	32.1	0.092
Urea (mg/dl)	40.7	37.8	11.5	0.564
T-bil (mg/dl)	0.26	0.29	0.12	0.679
ALP (U/l)	235	279	75.8	0.186
CPK (U/l)	465	167	229	0.076
LDH (U/l)	453	250	214	0.022
AMY (U/l)	12.9	9.19	6.56	0.211
LIPA (U/l)	29.1	15.8	10.5	0.326
ALT (U/l)	5.26	6.62	3.88	0.421
AST (U/l)	237	295	117	0.258
γ-GT	112	129	81.1	0.624
Ca (mg/dl)	11.9	13.4	1.73	0.049
Zn (mg/dl)	74.9	87.2	45.3	0.529
Cu (μg/dl)	116	133	27..9	0.158
Na (mmol/l)	169	169	0.83	0.228
Fe (μg/dl)	30.9	33.1	10.8	0.630
P (mg/dl)	4.07	5.67	2.21	0.110
Mg (mg/dl)	2.50	2.71	0.57	0.405
Chloride (mmol/l)	100	103	11.9	0.510
α-TOH	2.11	1.87	0.61	0.785

**Note:**

GLU, glucose; TG, total triglycerides; TP, total protein; TC, total cholesterol; T-bil, total bilirubin; ALP, alkaline phosphatase; CPK, creatin-phospho-kinase; LDH, lactic dehydrogenase; AMY, amylase; LIPA, lipase; ALT, alanine-amino transferase; AST, aspartate-amino-transferase; γ-GT, gamma-glutamyl transferase; Ca, calcium; Zn, zinc; Cu, copper; Na, sodium; Fe, iron; P, phosphorus; Mg, magnesium; α-TOH, alfa-tocopherol.

**Table 2:** Hotelling’s test was non significant. Assignment of individuals according to coat color, based on the metabolic profile, following the discriminant analysis of blood serum metabolite levels.

Observations and percentage based on coat color			
Coat	Albino	Pigmented	Total
Albino	8	0	8
	100.00	0.00	100.00
pigmented	0	17	17
	0.00	100.00	100.00
Total	8	17	25
	32.00	68.00	100.00
*P*-value < Mahalanobis quadratic distance			0.440

The circulating levels of α-TOH in the blood serum displayed to vary non significantly in all animals of both breeds (Table 1).

Table 3 reports the standar canonical coefficients obtained at the CANDISC based on the multivariate analysis among 22 correlated metabolites into linear uncorrelated new coefficients. The highest absolute value of calculated coefficients (CCs) indicate which variables out of the totality better describe the breed specific metabolic profile, out of the complete set of metabolites analyzed. It can be seen that UREA, total protein, total triglycerides, Mg and Zn can have the major effect in discriminating individuals according to the coat, whereas the least values of coefficients are attributed to Fe, Glu, Ca, ALP and P.

**Table 3:** Standardized canonical coefficients obtained at the CANDISC, based on the multivariate analysis among 22 correlated metabolites into linear uncorrelated new coefficients. Assignment of individuals is according to coat color, based on the metabolic profile.

Standardized canonical coefficients of total sample	
Variables	Can1
LDH	10.65128556
GLU	−8.21006919
NA	12.76166681
Chloride	−14.96052237
TG	−26.32997950
FE	8.81602440
P	−2.59032922
MG	25.52399813
CK	11.70253834
CA	7.55956610
ALP	−4.91432391
ZN	−23.39275922
CU	−11.11046095
TP	31.10549327
UREA	−39.84895236
AMY	14.33844090
LIPA	−21.13379349
TC	18.00655403
ALT	32.99963711
AST	11.95529756
T-BIL	8.04716134
γ-GT	15.07024699

## Discussion

Albinism in wild animals may represent a serious health risk under excess of sun exposure (Prado-Martinez et al., 2013). By contrast, the description of the presence of Asinara donkeys on the homonymous island dates back to the 18th century (Cetti, 1774). Despite the critical status of conservation, it could be reasonably postulated that alternative solutions to UV damage and oxidative stress may help to explain the adaptation to the Mediterranean environment of OCA1 albino donkeys. The comparative approach used to explore the metabolic profile of specimens of Asinara vs. Sardo breeds pointed to unequivocal results when the DA analysis was carried out on 22 metabolites analyzed for each individual. This is suggestive of the fact that the metabolic profile of donkeys determined during the month of June (which represents the peak of the year when the light hours per day reach the maximum) may represent the natural stressing condition when albino animals should display to adapt to environmental conditions. Indeed, the sharp though non significant assignment of individuals to own respective breed, based on the metabolic profile, seems to point to this explanation. The CANDISC analysis revealed that a reduced number of metabolites can efficiently summarize the differences between the metabolic profile observed in individuals of the two breeds. Urea in the bloodstream, involved in the metabolism of nitrogen, is one of those. Urea is a peculiar form of nitrogen utilization, strongly linked to the amount of circulating fluids. In most of mammals, Urea is formed mainly from proteins (endogenous/tissue and exogenous/feed). Together with Urea, also TP contribute to depict the metabolic profile of individuals belonging to the two breeds, followed by TG, Mg and Zn. Combined together, such metabolites may seem to point to peculiar protein turnover, energy and metabolic activity according to the two breeds. It is interesting to point out, however, that blood serum concentration of such metabolites does not display to differ significantly between the two breeds.

Melanin represents the natural pigment of skin, hair, iris and natural opens, produced by melanocytes to protect tegument cells from UV damage. To such an extent, albinism in wild and feral animals may represent a conditioning factor behind the environmental adaptation as well as a potential driver of the selection of feed sources rich in antioxidant compounds. A possible strategy for albino grazers in the wild may consist in an increased intake of feeding sources rich in natural antioxidants, like α-TOH. However, the vitamin E status found in this trial turned out to differ not significantly between individuals of different breeds.

There is general consensus of the correlation between circulating values of α-TOH below adequacy (2 μg/ml) and higher risk of myopathy and neurodegenerative disorders for the horse, though a linear effect on the onset of clinical signs is still under debate (Cummings et al., 1990; Blythe & Craig, 1992; Divers et al., 1992; Hahn, Mayhew & Shepherd, 1993; Sustronck et al., 1993; Kuwamura et al., 1994; Landolt, Feige & Grest, 1997; Finno et al., 2016). No specific health problems were found in Asinara donkeys if compared to Sardo donkeys under same grazing conditions. In general, wild animals are aware grazers in nature and can wisely select feeding sources to avoid toxic or harmful plant species. Captive animals or domestic livestock may be untrained to anti-pastoral or anti-nutritional traits of plants in natural pastures (Aboling et al., 2016). Potential xenobiotic ingestion leading to photosensitization in Asinara donkeys cannot be excluded but represents a remote explanation to skin redness and epiphora observed.

The variation of circulating levels of LDH and CPK in the two breeds of donkeys seems to figure out a different underlying health condition, because when those enzyme increase in the blood serum, this necessarily points to cell damage. The association between the two, being higher in the Asinara donkeys solely, may be suggestive of some damage at muscular tissue level, though no clinical effects could be observed relating to any specific factor, nor attributable to the syndrome from deficient vitamin E, like described in the literature for the horse. In fact, no similar conditions in the donkey were described before. However, the hypothesis that albino donkeys may be prone to metabolic perturbation can be here supported by the increase of those enzymes found exclusively in apparently healthy Asinara donkeys, against the background of comparable circulating levels of α-TOH found in both breeds.

## Conclusions

Results obtained in this study point to differences in the metabolic profile of Asinara vs. Sardo donkeys, freely grazing at Mediterranean latitude at the peak of positive photoperiod. Interestingly, the analysis of the biochemical profile based on 22 parameters explored in this trial allowed to observe an unequivocal assignment of individuals to own respective breed, though variations of circulating levels turned out to be non significant (probably due to the low number of albino specimens). The vitamin E status appeared to be similar in both breeds, being slightly higher in the albino donkeys. Worth of note, the increased levels of LDH and CPK enzymes were found in apparently clinically healthy animals. In view of such high levels in the bloodstream of Asinara donkeys, skeletal myocyte condition deserves detailed investigation which may provide evidence for a higher cell membrane instability.

## Supplemental Information

10.7717/peerj.9297/supp-1 Supplemental Information 1 Raw data.

## Additional Information and Declarations

### Data Availability

The following information was supplied regarding data availability: Raw data are available as a Supplemental File.
